# From Attire to Assault: Clothing, Objectification, and De-humanization – A Possible Prelude to Sexual Violence?

**DOI:** 10.3389/fpsyg.2017.00338

**Published:** 2017-03-10

**Authors:** Bhuvanesh Awasthi

**Affiliations:** ^1^Institute of Neuroscience and Psychology, University of GlasgowGlasgow, UK; ^2^Epistemic ConsultantsNew Delhi, India

**Keywords:** clothing, objectification, object perception, dehumanization, sexual violence

## Abstract

In the context of objectification and violence, little attention has been paid to the perception neuroscience of how the human brain perceives bodies and objectifies them. Various studies point to how external cues such as appearance and attire could play a key role in encouraging objectification, dehumanization and the denial of agency. Reviewing new experimental findings across several areas of research, it seems that common threads run through issues of clothing, sexual objectification, body perception, dehumanization, and assault. Collating findings from several different lines of research, this article reviews additional evidence from cognitive and neural dynamics of person perception (body and face perception processes) that predict downstream social behavior. Specifically, new findings demonstrate cognitive processing of sexualized female bodies as object-like, a crucial aspect of dehumanized percept devoid of agency and personhood. Sexual violence is a consequence of a dehumanized perception of female bodies that aggressors acquire through their exposure and interpretation of *objectified* body images. Integrating these findings and identifying triggers for sexual violence may help develop remedial measures and inform law enforcement processes and policy makers alike.

## Introduction

A myriad of issues in body and object perception, agency attribution and de-humanization highlight the centrality of psychological science in understanding how individuals become involved in violence, particularly sexual violence, in human society. In the recent past, several editorial and opinion articles published in popular news media have discussed the issue of sexual assault in the context of clothing and women’s attire. When a series of articles open up to public discourse the question of how women’s attire is relevant to sexual assault, it seems pertinent to go a step further and examine the neuroscientific research on body perception and objectification. This is especially important when there is a relative paucity of research connecting the dots to offer a thoughtful and comprehensive framework within which to examine the issue. Arguments in this cross-cutting perspective article offer new insights into the issue of sexual violence in human society. The aim of this article is not to dispute any existing issues under debate in the research literature (see Thornhill and Palmer, 2000; Kimmel, 2003; Palmer and Thornhill, 2003; McKibbin et al., 2008). Instead, I intend to review additional neuroscientific evidence in this context that highlights linkages between visual perception, attire and objectification. Integrating these findings may help develop remedial measures and solutions to prevent and respond to sexual violence.

## Clothes and the Perception of Human Bodies

Humans are a large, heterogenous social group and perform different functional roles as the members of a society. Clothing serves an important socializing influence and acts as a symbol of social status and identity (Kaiser et al., 2001). Clothing plays a crucial role in the identity politics of urban societies. Doctors, nurses, soldiers, police and military men, postmen (and many other public servants), advocates and judges, priests and the pope, politicians, comedians, actors (and other entertainers) are all identified and called upon by their attire. Clothing is also informative versus un-informative. A deviation from the norm makes the attire informative. The concept of uniform is a non-deviation from the norm and leads to uniformity within a social context.

The act of constructing one’s identity is not an abstract metaphysical one, it is very much about the veneer, the persona. Dressing can be an act of making the self available to others, not only for appreciation and admiration, but also for objectification. Clothing reflects the self — the identity, the material practice we engage with in daily life (Lynch, 2007; Woodward, 2008). One’s wardrobe is known to be an extension of the diverse aspects of one’s beliefs and constructs social identity (Hill, 2005; Woodward, 2008). In this process of identity creation, there is an attempt to strike a balance between the dynamic interplay of conformity and individuality – identified as a core aspect of fashion (Simmel, 1971).

How animals and humans recognize their conspecifics for the purposes of detecting threat (to avoid) and potentially mate (to attract/approach) is a topic of intense ongoing research. Essential amongst external cues are face and body perception, body movement, shape and appearance to assess signals of threat, fecundity or other cues. Faces and bodies provide a vast array of social cues that are relevant for perception and communication. Rapid assessment of identity, gender, age, intentions, and emotional state is made through faces as well as bodies. Scores of research findings have established that bodies (as well as faces) are perceived by separate brain mechanisms than those for inanimate objects (Kanwisher and Yovel, 2006; Peelen and Downing, 2007). Bodily cues provide crucial information for people perception and toward understanding their emotions and intentions (de Gelder, 2006).

Over the course of time as humans developed socio-cultural practices, reorganizational shifts are likely to have occurred in cognitive and perceptual mechanisms. Since humans do not have direct access to others’ minds, they have to use external cues to infer the mental states of others [e.g., intentions, beliefs, desires (Carruthers and Smith, 1996)]. The ability to attribute mental states to others is called theory of mind (Premack and Woodruff, 1978) or mentalizing (Frith and Frith, 2003). Attributing mental states to others is essential for understanding others and for developing social communication and empathy.

Barring some tribes in remote locations, humans are distinct from other animals in that they cover their bodies with clothes (or leaves and other natural or artificial items). Both Neanderthals and prehistoric *Homo sapiens* are believed to have covered themselves with some sort of clothing. Humans likely started wearing clothes as early as 170,000 years ago (Toups et al., 2011). Clothing, symbols and other paraphernalia that signal social status, intentions and other relevant communication increasingly mediated recognition of humans in a social context.

What prompted humans to begin covering their bodies is a question that requires more research, both in the cognitive neuroscience and sociology domain. It is likely that weather patterns, moving away from inhabitation in the wild toward agriculture and domestication processes, covert (concealed) ovulation, and a year-around period of fecundity (mating) coupled with sexual swelling of genitalia showing arousal played some role in the development of human clothing. In a recent study by Street et al. (2016), sexual swellings in female non-human primates were shown to be reliable signals of female fertility.

Forced copulation is documented in several animal species (Lalumière et al., 2005) and it might be useful to examine its occurrence in humans. In this article, however, the focus is on de-humanization and objectification, both factors that likely contribute to sexual assault and violence. Are humans perceived as sexually autonomous or as sexual objects? What factors are likely to lead to sexual objectification? To address a pressing political and public safety imperative, and to develop an empirically informed understanding of the causes, risk factors, and management strategies related to sexual violence, it is essential to examine issues in objectification research and the neuroscience of person perception.

## Body Perception, Sexual Objectification, and Denial of Agency

According to objectification theory (Fredrickson and Roberts, 1997), female bodies are scrutinized and evaluated to a greater degree than male bodies, leading to sexual objectification of women. The objectifying gaze is known to occur in interpersonal encounters and media representations of women (Fredrickson and Roberts, 1997; Kilbourne and Jhally, 2000). In recent years, psychologists have investigated the objectification processes in detail. Provocative clothing that leads to deviation from routine modesty norms approaches objectification. Kennedy (1993) defines provocative dress as clothing that deviates from the norm by alluding to a more sexually charged context than the one in which it is worn. Note that the emphasis is on the *margin* of acceptability.

A number of studies have examined the objectification of bodies in the context of whether they were covered or uncovered. It was found that when wearing underwear or a swimsuit, a person could be viewed as a mere body that exists for the pleasure and use of others (Bartky, 1990). Other studies found that swimsuit-wearing women expressed more body shame and performed worse on a math test than did sweater-wearing women (Fredrickson et al., 1998). Sexual objectification has been related to decreased mind attribution (Loughnan et al., 2010) and diminished agency perception (Cikara et al., 2011). Sexualized women are perceived as less competent and less fully human (Vaes et al., 2011).

A focus on appearance rather than on personality diminished the degree of human nature attributed to females (Heflick and Goldenberg, 2009). The recognition and attribution of human nature is key to social perception, allowing people to differentiate humans from objects (Loughnan and Haslam, 2007). However, it is not only men who dehumanize sexualized women and their representations. Widespread beliefs that women are sex objects are shared by both men and women at a basic cognitive level (Gervais et al., 2011, 2012; see also Heflick et al., 2011). When women sexualize their appearance, they are at a far greater risk than men. A focus on appearance, instead of personality, increased the objectifying gaze toward women, as demonstrated by increased eye movements toward their chests and waists compared to their faces (Gervais et al., 2013b).

Women are also known to self-objectify when they choose clothes for fashion over comfort (Tiggemann and Andrew, 2012). For example, aerobics and ballet participants wearing tight-fitting outfits generated greater negative feelings toward their bodies, selves and performance, compared to those wearing loose-fitting outfits (Price and Pettijohn, 2006). Higher levels of self-objectification have also resulted in unipolar depression, sexual dysfunction, and eating disorders (McNelis-Kline, 2000; Prichard and Tiggemann, 2005). When put in a self-objectifying situation (such as wearing a one-piece, Speedo bathing suit), both men and women of all ethnicities experienced negative outcomes. Sexual objectification has more adverse consequences for women than for men (Moradi and Huang, 2008; Saguy et al., 2010; Gervais et al., 2011), affecting mental health, intellectual performance and increasing the risk of depression (Jack, 1991; Whiffen et al., 2007). Objectification also tends to make women behave as lesser beings in social interactions (Saguy et al., 2010).

Haslam and Loughnan (2014) provide empirical support to explain the differentiation of humans from animals and robots, and demonstrate that such differences are convergent across cultures. Humans are distinct from animals in possessing and developing unique attributes like cognitive capacity, civility, and refinement. Similarly, due to emotionality, vitality and warmth, the quality of humanness is different from inanimate objects (Haslam and Loughnan, 2014). Drawing from social cognition research, Harris and Fiske (2006) argue that dehumanization is the failure to spontaneously consider another person’s mind. In the neural context, it is said to de-activate the social cognition network, specifically cortical regions such as the medial prefrontal cortex and superior temporal sulcus.

Through a series of experiments, Vaes et al. (2011) demonstrate that only objectified women were associated with less human concepts. The authors further show that sexually objectified women shift a man’s focus toward a female target, away from her personality and more onto her body, triggering a dehumanization process. In contrast, women dehumanize sexually objectified women by distancing themselves from the sexualized representations of their own gender category. Another recent study points out that the perception of sexualized women deploys cognitive mechanisms specific to object perception, while sexualized men are perceived as persons (Bernard et al., 2012; also see Bernard et al., 2013; Tarr, 2013; Schmidt and Kistemaker, 2015). Specifically, Bernard et al. (2012) showed that sexualized female bodies are perceived as objects.

Previous work has demonstrated that persons (faces and bodies) are recognized in an upright condition more easily, compared to when inverted. In contrast, objects don’t seem to manifest this inversion effect. Bernard et al. (2012) showed that sexualized female bodies are recognized in an inverted manner quite easily, comparable to object-like cognitive processing. Further, Gervais et al. (2012) demonstrate that akin to object parts, sexualized body parts can be recognized easily without the entire body context. Gervais et al. (2012) show that sexualized females are interchangeable with objects. Further, Bernard et al. (2015a) examined factors that may prompt more configural processing and less objectification of sexualized female bodies. Replicating previous findings showing that sexualized female bodies, but not sexualized male bodies, elicited less configural processing and more objectification, the authors demonstrate the salience of sexual body parts as a crucial determinant of object-like part-based analytic processing of sexualized female bodies. However, when body features were masked and humanizing information was provided—female bodies were processed configurally, indicating the possible plasticity of cognitive objectification of women.

## Media Images and Objectification

Besides social groups, peers and families, media images of women are one of the primary culprits in teaching girls to self-objectify (Kilbourne, 1994; Kilbourne and Jhally, 2000). Images from television, video games, films, magazines, and many other sources disproportionately use female bodies to hock products, and the camera frame often focuses on female body parts rather than the whole picture in an objectifying manner (Archer et al., 1983; Kilbourne, 1994). Roberts and Gettman (2004) suggest that mere exposure to objectifying media plays a significant role in the initiation of a self-objectified state along with its attendant psychological consequences for women. Within a broader context, Levy (2005) discusses the emergence of ‘raunch culture’ highlighting that much of commercially marketed sexual liberation imagery of women actually reinforces the sexual objectification of women.

Peter and Valkenburg (2007) describe that there is increased sexual content in the media, and the sexualized portrayal of women in advertisements went up significantly between 1983 and 2003 (Reichert and Carpenter, 2004). As stated previously, exposure to sexually objectifying media has been linked to self-objectification, body shame, anxiety over appearance, and an acceptance of the normative belief that women are sexual objects (Ward and Friedman, 2006; Peter and Valkenburg, 2007). Lynch (2007) showed that body-revealing behaviors, when practiced within social contexts, served to reinforce patterns of sexual objectification of women rather than an expression of female sexual agency (also see Levy, 2005). Grabe and Hyde (2009) report that self-objectification is mediated through a direct relation between music television (MTV) viewing and body esteem, dieting, depressive symptoms, anxiety, and confidence in mathematics ability in female viewers (also see Harrison and Hefner, 2006; Grabe et al., 2008).

Females are portrayed as sex objects in a vast majority of magazine advertisements targeted both at men and women (Lindner, 2004; Baker, 2005; Stankiewicz and Roselli, 2008). Halliwell et al. (2011) show that contemporary objectified media portrayal of women has a powerful and problematic impact on psychological well-being and disordered eating behaviors, in particular weight concern and self-objectification. As is the case with adult women, Tiggemann and Slater (2015) provide further evidence that mere magazine and Internet exposure and appearance conversations with friends predicted self-objectification in early adolescent girls, which in turn causes body shame, dieting and depressive symptoms, in accord with the pathways postulated by objectification theory.

Widespread normalization of women-as-bodies in modern culture derives from the use of their bodies in advertising and entertainment (Kilbourne and Jhally, 2000; also see Conley and Ramsey, 2011). Alongside, chilling cases of sexual offenses are rampant across nations with increasing incidence (United Nations, 2015), pointing to a deeper, underlying issue of the objectifying gaze, aided and perpetuated globally through certain media representations of women and interpersonal encounters. Exploring if cultural background may have modulatory effects on rape perception, Loughnan et al. (2015) examined the objectification of others and self-objectification in seven countries (Australia, India, Italy, Japan, Pakistan, UK, and USA). The authors report that objectification of others and self-objectification was more acute in Australia, Italy, UK, and USA compared to Japan, India, and Pakistan. In the lure of importing cultural cues from “relatively progressive western societies” (The Hindu, 2012, Editorial), opinion-building media fail to focus on the pervasive nature of objectification. Sexual objectification of women is encouraged, promoted and socially sanctioned through a variety of ways, including, but not limited to, beauty pageants, cheerleading, and cocktail waitressing (Moffitt and Szymanski, 2011; Szymanski et al., 2011). A study on gender bias highlights the problematizing of the female body in films across 11 countries (Smith et al., 2014), putting India amongst the top.

The female body gains attention and is evaluated against unrealistic ideals that are often sexualized (Fredrickson et al., 2011). Women learn to portray themselves as objects on display, believing their appearance determines their value (Moradi and Huang, 2008; Szymanski et al., 2011; Smith, 2015). Kane et al. (2013) showed that sexualized portrayal of female athletes leads to negative objectification, resulting in making them feel more like sex objects, rather than being proud of their athletic capacity and success. Smith (2015) further confirms that women who view glamorized and sexualized images were more likely to self-objectify using body and appearance descriptors. Examining the degree and nature of sexualization in girls’ clothing, Goodin et al. (2011) reported substantial presence of sexualization in girls’ clothing being marketed to and worn by young girls in the US, thereby contributing to the cultural objectification of pre-teen girls. The authors report covert, ambiguous sexualizing in clothing that makes objectification more complex. In a recent study, Galdi et al. (2014) demonstrated that males exposed to objectifying TV (where women were portrayed as sexual objects) reported greater likelihood of engaging in sexual coercion and sexually harassing behavior, compared to conditions were men were exposed to professional portrayal and neutral content.

## Consequences of Dehumanization: Prelude to Violence

As stated earlier, humans are distinguished from animals on attributes involving cognitive capacity, civility, and refinement, as well as from inanimate objects on the basis of emotionality, vitality, and warmth (Haslam and Loughnan, 2014). Contrary to the belief that everyday forms of dehumanization are innocent and inconsequential, Kristoff (2014) has argued that the evidence reveals profoundly negative consequences for both victims and perpetrators. Dehumanization, the denial of agency and personhood contributes to large-scale intergroup conflict and violence (Haslam and Loughnan, 2014; Waytz et al., 2014). Dehumanization as a consequence of sexual objectification has dire consequences. Loughnan and Pacilli (2014) distinguish the consequences of objectification along the attitude/behavior distinction, highlighting an important aspect that has received less research attention.

On the attitudinal front, sexually objectified humans are likened to objects or automata with no capacity for qualities such as warmth, emotion, and individuality (Haslam, 2006). Cikara et al. (2011) report that viewing sexualized images of women reduced brain activation in areas for mental state attribution, while Vaes et al. (2011) showed that sexualized women are implicitly associated with animals by both male and female perceivers. Milburn et al. (2000) examined perceptions of rape in an ethnically and socioeconomically diverse sample. The authors reported that those males who viewed sexually objectifying R-rated films reported diminished view of the victim’s suffering and thought the victim deserved sexual assault. In a sexually objectified context, the target’s clothing increased victim blaming and lower moral concern (Workman and Freeburg, 1999; Grubb and Harrower, 2009) in an acquaintance rape circumstance (Loughnan et al., 2013), highlighting animalization and infra-humanization as a result of clothing and objectification. Another recent study examining the influence of sexual objectification on men and women’s rape perceptions, Bernard et al. (2015b) show that sexual objectification increased victim blaming and diminished rapist blame in cases of stranger rape.

Both objectification and infra-humanisation make women vulnerable to violence. Similarly, research literature on the topic has established the sexualisation-to-meat link (Adams, 1990) wherein the denial of emotionality and agency reduces animals to meat producing units. The capacity of the animal to suffer is perceived to be significantly less when the animal is perceived as food (Bratanova et al., 2011). Cruelty laws are differentially applied to pet and farm animals due to this distinction. Bongiorno et al. (2013) have argued that objectification results in reduction of human attributes to sexualised women and experimentally demonstrated that using sexualized images of women reduces support for ethical campaigns.

On the behavioral side, a number of studies (Bargh et al., 1995; Mussweiler and Förster, 2000; Landau et al., 2004; Gruenfeld et al., 2008) describe a complex interplay between power, sex, and aggression that might lead to violence toward presumed sexually appealing and available women. Some early research has shown that objectified women are subject to sexual harassment, sexual coercion and unwanted sexual attention (Fitzgerald et al., 1988), especially following exposure to objectifying media (Rudman and Borgida, 1995; Galdi et al., 2014) in public spaces by strangers (Fairchild and Rudman, 2008). Men with hostile and aggressive views toward women are more likely to objectify. Further, attribution of animalistic lack of agency and reduced pain attribution results in higher likelihood of violence toward objectified targets. Rudman and Mescher (2012) demonstrate that men who implicitly associate women with animals and objects have a higher propensity for sexual aggression. Figueredo (1992) and Thornhill and Palmer (2000) have argued that males who commit rape are likely to have psychopathologies, social inadequacies, experience of childhood sexual trauma, lack of social competence and empathy (Stermac and Quinsey, 1986; Lipton et al., 1987; Lalumière et al., 2005). Sexual assaulters are similar to other violent offenders and tend to have extensive non-sexual criminal histories. Examining the impact of objectification in the domain of sexual assault, Loughnan et al. (2013) found that an objectified woman is blamed herself for being raped and is perceived to suffer less.

Dehumanization also underlies maltreatment and violence toward ethnic or racial minorities and animals. Reduced mind-attribution (as a result of dehumanization) makes it easier for the perpetrator to deny pain and agency to the dehumanized group. Research findings describe increased violent behavior, harsh treatment and reduced empathetic concern toward dehumanized targets (Zebel et al., 2008; Cehajic et al., 2009; Viki et al., 2013). It seems predictable that sexual violence is a consequence of a dehumanized perception, particularly of female bodies and a generalized antisocial trait that aggressors acquire through their exposure and interpretation of body images. Providing additional evidence for the mediating role of objectification in sexual violence, Gervais et al. (2014) report that heavy drinking was associated positively with sexual objectification and sexual violence perpetrated by men.

Highlighting the sexual signaling function of clothing, Jeffreys (2005) points out that female clothing items also emphasize women as sexual objects aligned to male desires. Goodin et al. (2011) describe for instance, a man’s professional attire (generally a suit) disguises his underlying body shape, while a woman’s professional attire comprises a more form-fitting suit with a skirt that shows her legs, accompanied by high-heeled shoes. Women in provocative clothing are rated as more flirtatious, seductive, promiscuous, and sexually experienced—and as less strong, determined, intelligent, and self-respecting (Koukounas and Letch, 2001; Gurung and Chrouser, 2007), emphasizing sexual availability and objectification. In contrast, Dunkel et al. (2010) demonstrate that young Muslim-American women wearing non-Western clothing and a head veil report significantly less pressure to attain the Western ideals of thin beauty, as compared to Muslim-American and non-Muslim women who wore Western-style clothing. Lahsaeizadeh and Yousefinejad (2012) describe the crucial interplay of attire in social contexts that determine the sexual harassment of women in public places.

## Epilog: Modified Perceptual and Cognitive Domains

Recognition of the human body in a social context is mediated by clothing and other social symbols. Body covering or attire became an integral part of creating a persona that is available for perception by others. Clothing is also one of the most significant indications of gender identity, even for very young children (Pomerleau et al., 1990; Barnes and Eicher, 1992). Clothing likely played a role in the signaling that may have previously been learnt through bodily cues. Cultivation theory suggests that exposure to repeated themes and images over time leads to the assimilation of those themes into a person’s world view (Gerbner et al., 1994). To understand what attire means, it is essential to explore both semiotics, as well as neuro-cognitive mechanisms. It does, however, point to how humans perceive and behave with their fellow members. Provocative dressing leading to sexual objectification biases the perception of sexual violence.

Certain cognitive domains can be formed or refined by interactions between neural mechanisms and learning exposure. Such exposures to cultural entrainment potentially recycle the neural resources otherwise utilized in more innately learnt biological behavior. Within the domain of higher-level visual learning, humans started to recognize and associate meaning with certain symbols (as in word reading) that occurred much later in evolution as opposed to body perception. For instance, in accordance with the neuronal recycling hypothesis, as a result of training to recognize and attribute meaning to symbols, a portion of the fusiform face area has been reallocated for word recognition (Dehaene and Cohen, 2007; Anderson, 2010). Given some early evidence emerging in this context (Bernard et al., 2012, 2013; Gervais et al., 2013a; Tarr, 2013), it is likely that clothed and unclothed body recognition started to diverge in line with cultural factors, sexual signaling and agency attribution. Bernard et al. (2012) show that nakedness and sexualized portrayals of female bodies shift the cognitive mechanisms from configural to more part-based analytical kind (more object-like as opposed to person-like), leading to objectification. Clothing may also play a likely role in the cognitive-emotional development of complex emotions such as shame, guilt and modesty. This article posits that perceptual domains for body perception are potentially modified following objectified learning, cultural entrainment and exposure to sexualized bodies. This sets the ground for further neuroscientific (experimental) investigations of the issue.

This article has potential limitation for wider aspect of sexual violence research. The present analysis does not dismiss the possibility that other mechanisms such as psychopathology (sexual disorders and personality disorders such as volitional impairment, Paraphilia; Quinsey et al., 1981; Miller et al., 2005) might also underlie sexual predation and violence. Instead, the focus here has been on connecting the links between attire, objectification and dehumanization that leads to sexual violence. The sexual subjectification, which is a result of women internalizing the judgmental male gaze on their body, results in deeper forms of social control, self-surveillance and inequality. Gill (2007) notes that modern media culture is preoccupied with the body, specifically, reducing women’s key identity to a ‘sexy body’ that is under constant monitoring, and judgment. Gill (2007) also argues that there is a shift in the portrayal of women as sex object to desiring sexual subject, wherein ‘women are not straightforwardly objectified but are presented as active, desiring sexual subjects who choose to present themselves in a seemingly objectified manner because it suits their liberated interests to do so.’ This, argues Gill, is a shift from an external, male judgmental gaze to an internal self-policing narcissistic gaze, resulting in a deeper form of exploitation than objectification. Highlighting the irony, such a representational shift from sexual objectification to that of assertive subjectification still denies women any humanized treatment and make them prone to attacks and vilification, with little opposition.

Combining research on objectification as well as on brain mechanisms of visual perception, the presented amalgam of findings adds perspectives from neuroscience research that is relevant to the topic and can help contextualize the discussion in greater detail. At the policy context level, there is a need to engage in a far larger effort to eradicate toxic attitudes, learnt consciously or unconsciously from the modern ‘schools’ of mass media (reality television, soap operas, cinema, and newspapers). Victimization might emerge, not only through prescriptive norms, with agents telling us *what not to wear* but also from those that cajole us *to wear* attire of certain kinds. Examining the narratives of clothing practices prevalent in the mass media, Jackson et al. (2012) highlight the contradictory discourses directed at pre-teens and teens, confusing, demeaning and repressing young women. As we develop new public and policy responses to sexual discrimination, harassment and assault, we need to re-examine some underlying psychological and biological processes. Integrating these findings and identifying triggers for sexual violence may help develop effective remedial measures. In addition to meaningfully contributing to existing knowledge, it is imperative to carry out more investigations in the future with prevention and intervention implications.

## Author Contributions

BA, at the University of Glasgow is the sole contributing author for this manuscript.

## Conflict of Interest Statement

The author declares that the research was conducted in the absence of any commercial or financial relationships that could be construed as a potential conflict of interest.
